# The effect of metronidazole plus amoxicillin or metronidazole plus penicillin V on periodontal pathogens in an in vitro biofilm model

**DOI:** 10.1002/cre2.96

**Published:** 2018-01-02

**Authors:** Gabriela Dabija‐Wolter, Sundus Saad Al‐Zubaydi, Marwan Mansoor Ali Mohammed, Vidar Bakken, Anne Isine Bolstad

**Affiliations:** ^1^ Department of Clinical Dentistry, Faculty of Medicine University of Bergen Norway; ^2^ Department of Clinical Science, Faculty of Medicine University of Bergen Norway

**Keywords:** Aggregatibacter actinomycetemcomitans, antibiotic combination therapy, antibiotic resistance, biofilm, periodontal, periodontitis

## Abstract

A combination of metronidazole (MET) and amoxicillin (AMX) is commonly used as adjunct to mechanical therapy of periodontal disease. The use of broad spectrum antibiotics such as AMX may contribute to development of antibiotic resistance. The aim was to evaluate the in vitro effect of replacing AMX with penicillin V (PV) in combination with MET on a biofilm model. A biofilm model consisting of Aggregatibacter actinomycetemcomitans, Porphyromonas gingivalis, and Fusobacterium nucleatum was developed. The biofilms were exposed to AMX + MET and PV + MET in two different concentrations. Bacterial viability in biofilms following antibiotic exposure was assessed by viable counts and by confocal microscopy. No live colonies of P. gingivalis nor F. nucleatum were retrieved from biofilms exposed to AMX + MET or PV + MET. The amount of A. actinomycetemcomitans was 4–5 logs reduced following antibiotic treatment; no statistical significance was achieved between AMX + MET or PV + MET treated biofilms. Replacement of AMX with PV at the same concentration, in combination with MET, resulted in similar effect on bacterial viability in this in vitro model. The option of using PV + MET instead of AMX + MET deserves further investigation, as this may contribute to reduce the risk of antibiotic resistance development.

## 1 INTRODUCTION

The loss of supporting tissues of teeth in periodontal disease is determined by the inflammatory response of the host to microorganisms in subgingival plaque. The depth of the periodontal pocket correlates with major changes in the composition of the subgingival microbial community, as this becomes more diverse and comprises a higher proportion of anaerobe species (Abusleme et al., 2013). Periodontal pathogens such as *Porphyromonas gingivalis* and *Aggregatibacter actinomycetemcomitans* possess a range of virulence factors that allow them to avoid and modulate the immune response of the host and thus persist at periodontal sites (Hajishengallis, Darveau, & Curtis, 2012). They might recolonize treated sites and cause recurrence of periodontal disease. *Fusobacterium nucleatum* facilitates biofilm formation of the periodontopathogens and late colonizers *P*. *gingivalis* and *A*. *actinomycetemcomitans* and supports their growth in biofilm, exhibiting mutualistic relationships with them (Ali Mohammed, Nerland, Al‐Haroni, & Bakken, 2013; Biyikoglu, Ricker, & Diaz, 2012; Periasamy & Kolenbrander, 2009). *A*. *actinomycetemcomitans* regulates protein expression of other species present in the biofilm (Bao, Bostanci, Selevsek, Thurnheer, & Belibasakis, 2015).

Mechanical debridement is the gold standard in the treatment of periodontal disease. However, in certain situations, an antibiotic treatment may be prescribed in order to eliminate or drastically reduce the amount of periodontal pathogens. The combination of amoxicillin (AMX) and metronidazole (MET) was initially adopted for treatment of patients with refractory periodontitis aiming to eliminate *A*. *actinomycetemcomitans* from the periodontal pockets (van Winkelhoff et al., 1989). Later studies have advocated the use of systemically administrated AMX + MET adjunctive to scaling and root planing for the treatment of chronic periodontitis, showing not only greater reduction in the levels of periodontal pathogens such as *P*. *gingivalis*, *Tannerella forsythia*, and *A*. *actinomycetemcomitans* but also improvements in clinical periodontal parameters (Haffajee, Patel, & Socransky, 2008; Sgolastra, Gatto, Petrucci, & Monaco, 2012; Sgolastra, Petrucci, Gatto, & Monaco, 2012; Soares et al., 2014). The combination of MET (targeting the bacterial DNA) with penicillin (targeting the bacterial cell wall) has synergistic effect and it was earlier recommended in order to increase bacterial susceptibility to antibiotic treatment (Baumgartner & Xia, 2003) (Khemaleelakul, Baumgartner, & Pruksakorn, 2002).

The growth of bacteria in biofilm favors the horizontal transfer of genes, including antibiotic resistance genes (Olsen, Tribble, Fiehn, & Wang, 2013; Roberts & Kreth, 2014). Overuse and misuse of systemic antibiotics have been related to the appearance or development of bacterial resistance in the subgingival flora (van Winkelhoff et al., 2000). In 2014, the Swedish health authorities introduced the use of penicillin V (PV) instead of the broad‐spectrum AMX in combination with MET as a contribution to reduce the risk of antibiotic resistance. The decision was based on knowledge of pharmacodynamics and pharmacokinetics of the antibiotics, knowledge of expected pathogens, risk for ecological damages, as well as clinical experiences (https://lakemedelsverket.se/antibiotikabehandling‐tandvard). Penicillin V has a narrow spectrum with low risk for adverse effects and disruption of the normal microflora. Risks for development of resistance drug interactions are low. Phenoxymethylpenicillin is on The World Health Organization's List of Essential Medicines, considered to be the most effective and safe medicines needed in a health system (https://en.wikipedia.org/wiki/WHO_Model_List_of_Essential_Medicines#Antibiotics).

The effect of combining MET and penicillin G (PG) on *A*. *actinomycetemcomitans* in vitro has been determined previously (Pavicic, van Winkelhoff, & de Graaff, 1992). However, to our knowledge, there is no in vitro study available to date investigating the effect of the MET + PV combination on periodontal pathogens existing in a biofilm.

The aim of this study was to assess the in vitro effect of replacing AMX with penicillin V (PV) in combination with MET, on a biofilm model consisting of *A*. *actinomycetemcomitans*, *P*. *gingivalis*, and *F*. *nucleatum*.

## 2 MATERIALS AND METHODS

### 2.1 Bacterial species and cultivation

*P*. *gingivalis* type strain ATCC 33277, *F*. *nucleatum ssp. nucleatum* strain ATCC 25586, and *A*. *actinomycetemcomitans* strain DSMZ 8324 were used in this study. Bacteria from frozen stock cultures were first grown on fastidious agar medium (FAA; Oxoid, Basingstoke, UK) for 48–72 hr, then transferred in liquid growth medium. Brain‐heart infusion (Difco Laboratories, USA) supplemented with hemin (5 μg/ml; Sigma‐Aldrich, Schnelldorf, Germany) and menadione (1 μg/ml; Sigma) was the liquid growth medium used in all experiments, referred to as BHI further in the text. Bacteria were precultured for 24 hr in liquid growth medium. Then an aliquot of the bacterial suspension was inoculated in fresh BHI and further incubated overnight and allowed to reach late log‐phase. All incubations were done in anaerobic conditions (5% CO_2_, 10% H_2_, and 85% N_2_) at 37°C (Anoxomat System, MART Microbiology, Lichtenvoorde, The Netherlands).

### 2.2 Biofilm construction

Biofilms were prepared in 24‐well plates (Corning Inc., NY, USA) by adapting a previously described protocol (Sanchez et al., 2011). Briefly, bacterial suspensions were prepared from overnight cultures in BHI. Bacterial concentration was adjusted by measuring optical density at 600 nm to 0.1 for all three bacterial strains. An aliquot from each bacterial suspension was then serially diluted and plated for checking the colony purity and number of colony forming units, CFUs. A bacterial mix was prepared in a sterile jar by pooling bacterial suspensions of *P*. *gingivalis*, *A*. *actinomycetemcomitans*, and *F*. *nucleatum* in volumes as 1:2:1. Each well was inoculated with 300 μl bacterial mix and 1,200 μl fresh BHI. The amount of bacteria in the inoculum were calculated as: 1.83 × 10^7^ ± 5.79 × 10^6^ CFU/ml *F*. *nucleatum*, 2.82 × 10^7^ ± 7.2 × 10^6^ CFU/ml *P*. *gingivalis*, and 4.43 × 10^7^ ± 1.44 × 10^7^ CFU/ml *A*. *actinomycetemcomitans* (mean and standard deviation).

The plates were incubated anaerobically to allow biofilm formation for 72 hr.

The biomass of the biofilm and the viability of bacteria were checked at different time points in preliminary tests in order to develop a mature biofilm for exposure to antibiotics. Bacterial mass in the biofilms was assessed by plate reading by plate reader (Synergy H1 Hybrid reader, BioTek instruments, USA). The biofilm biomass absorbance was measured at 600 nm immediately after complete growth medium removal.

### 2.3 Bacterial viability in biofilm

The viability of bacteria in biofilm was assessed by culture plate counting of CFUs. First, the growth medium and planktonic cells were removed from the 3‐day old biofilms, each biofilm was scrapped off the well by use of a sterile cell scraper (Sarstedt, Newton, USA), suspended in 1 ml fresh BHI, and disrupted by vigorous pipetting. The biofilm suspensions were 10‐fold diluted on FAA plates in order to obtain plates containing CFUs in range of 1–500 colonies. After 5–7 days of incubation in anaerobic conditions, CFUs were enumerated on FAA plates by help of a stereomicroscope, distinguished by the colony morphology. All experiments were run in triplicate.

The biofilm development and structure were assessed by confocal laser scanning microscopy (CLSM). Prior to examining under CLSM, the biofilms grown for 3 days on 4‐wells chamber slides were stained with FilmTracer^™^ LIVE⁄DEAD Biofilm Viability kit (Molecular Probes BV, Leiden, The Netherlands). Fully hydrated biofilms were stained for 20–30 min in the dark, in anaerobic conditions, thereafter immediately examined by CLSM (Leica TCS SP5; Leica Microsystems, Germany) equipped with an oil‐immersion of 63× or 100× objective (Carl Zeiss, Jena, Germany) with beam path settings at 488 nm and 543 nm, respectively. Fluorescence intensity thresholds were manually set for each fluorescent color when examining a negative control biofilm and not further modified. At least three different areas on each biofilm were investigated. Image stacks were analyzed with the Leica Confocal Lite^®^ software (Leica Microsystems).

### 2.4 Biofilm assays

Antibiotic cocktails to be used in assays were freshly prepared in BHI prior to each experiment. The antibiotic concentrations tested in this study were defined as “high concentration” when combinations used were AMX + MET (1,080 μg/ml + 2,160 μg/ml) and PV + MET (1,080 μg/ml + 2,160 μg/ml); and “low concentration” when AMX + MET (360 μg/ml + 720 μg/ml), respectively, PV + MET (360 μg/ml + 720 μg/ml) were used.

Growth medium together with planktonic bacteria were carefully removed and antibiotic solutions in BHI at given concentrations were gently placed in each well, in order not to induce biofilm disruption. Biofilms exposed to chlorhexidine (CHX) 0.2% (Corsodyl, GlaxoSmeethKlein, UK) were used as positive control. Fresh BHI alone was used for the negative controls. The challenged biofilms in 24‐well plates were incubated anaerobically for 2 hr. Then, the growth medium containing antibiotics or CHX were removed and each biofilm was harvested in 1 ml fresh BHI and disrupted as described above. Viable counts were recorded on FAA plates incubated for 5 days.

### 2.5 Statistical analysis

Paired *t* test was used to investigate significant differences between the two different combinations of antibiotics. A *p* value less than .05 was considered statistically significant.

## 3 RESULTS

### 3.1 Biofilm formation

**Figure 1:**
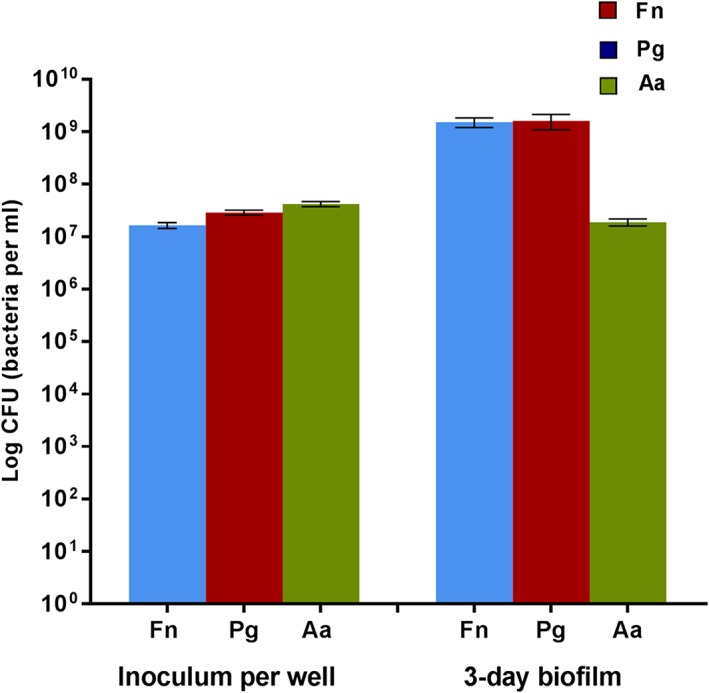
Viable counts of each bacterial species present in the initial inoculum (per well) and in the 3‐day old biofilms (*n* = 7). Fn = *Fusobacterium nucleatum*; Pg = *Porphyromonas gingivalis*; Aa = *Aggregatibacter actinomycetemcomitans*. Error bars: standard error

All three types of bacterial strains were harvested from 3‐day old biofilms. The viable bacteria in biofilms were mainly represented by *F*. *nucleatum* and *P*. *gingivalis,* which showed a nearly two‐log increase in numbers during the 3 days incubation. Viable counts of *A*. *actinomycetemcomitans* declined in the biofilm compared to the initial inoculum per well, as depicted in Figure 1. The bacterial mass of the 3‐day old biofilm as measured by plate reading (*n* = 7) was 0.568 ± 0.075 AU.

**Figure 2:**
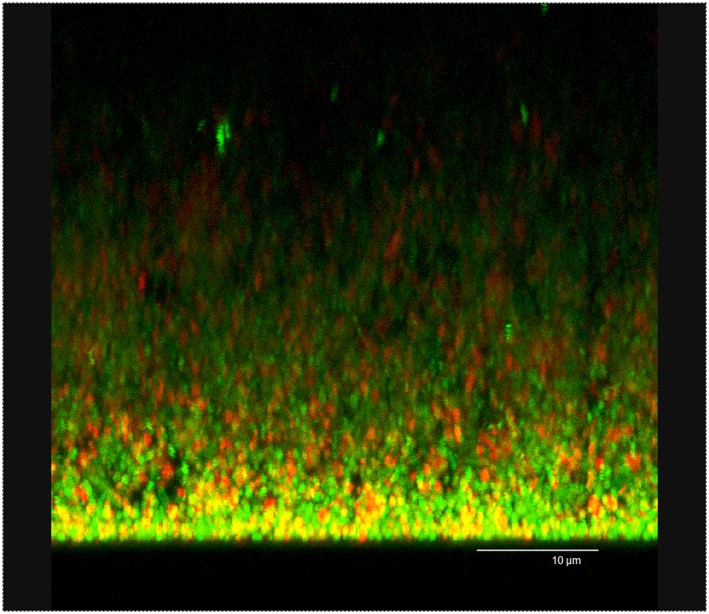
CLSM image in maximum projection of the series taken in xzy axis of the 3‐day old biofilm. Viable and nonviable bacterial cells are depicted in green and red, respectively. Scale bar: 10 μm

A mature biofilm with three‐dimensional structure with mushroom‐like shapes and nutrient channels was formed after 3 days incubation in anaerobic conditions. Both live and dead bacterial‐like structures could be observed by CLSM as shown in Figures 2 and 4a.

### 3.2 Effect of antimicrobials on biofilms

Exposure of biofilms to CHX for 2 hr resulted in eradication of all three bacterial strains, as no CFU could be detected at plate counting. A shorter exposure time than 2 hr to CHX 0.2% resulted in incomplete elimination of live bacteria from the biofilms; while exposure for 2 hr to CHX 0.2% diluted in 1:2 or 1:4 volumes resulted also in detection of CFU after harvesting of positive‐control biofilms (results not shown).

**Figure 3:**
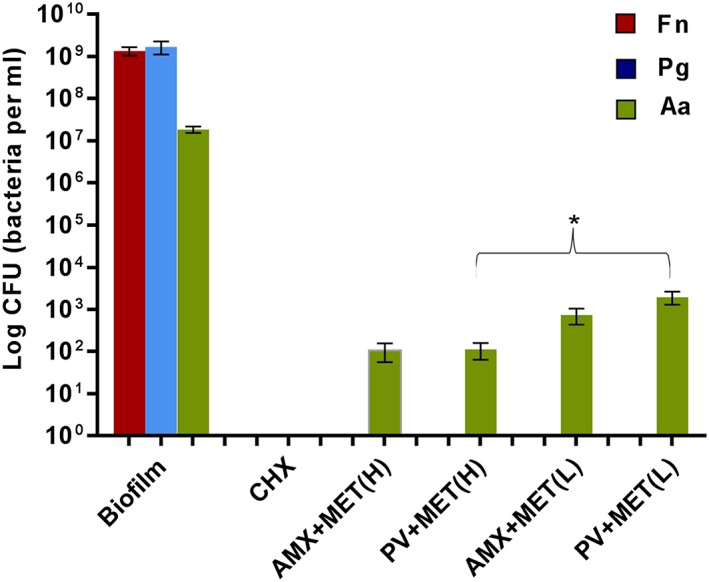
Viable counts of each bacterial species in the 3‐day old biofilms (negative controls) and subjected to antiseptic or antibiotic treatment (*n* = 6). No live CFU were retrieved from biofilms treated with CHX (positive controls). Single green bars: number of live *Aggregatibacter actinomycetemcomitans* retrieved from biofilms exposed to antibiotic combinations in high (H) or low (L) concentrations. * shows statistical significance between biofilms exposed to PV + MET in high and low concentration (*p* = .041, *t* test). Fn = *Fusobacterium nucleatum*; Pg = *Porphyromonas gingivalis*; Aa = *A*. *actinomycetemcomitans*. Error bars: standard error

Both *F*. *nucleatum* and *P*. *gingivalis* were completely eliminated from the biofilms by antibiotic treatment, both AMX + MET and PV + MET in high and low concentrations, as presented in Figure 3. The amount of *A*. *actinomycetemcomitans* was five logs reduced following exposure of biofilms to high concentration of antibiotics and four logs following low concentration. There was no statistical difference (paired *t* test) between the AMX + MET and PV + MET combinations with respect to growth inhibition of *A*. *actinomycetemcomitans* neither in the high concentration (*p* = .95) nor in the low concentration (*p* = .17). The combination PV + MET in high concentration had significantly stronger effect in eliminating *A*. *actinomycetemcomitans* than the low concentration (*p* = .041). No significance was achieved when comparing between the effect of low and high concentrations of AMX + MET (*p* = .076).

**Figure 4:**
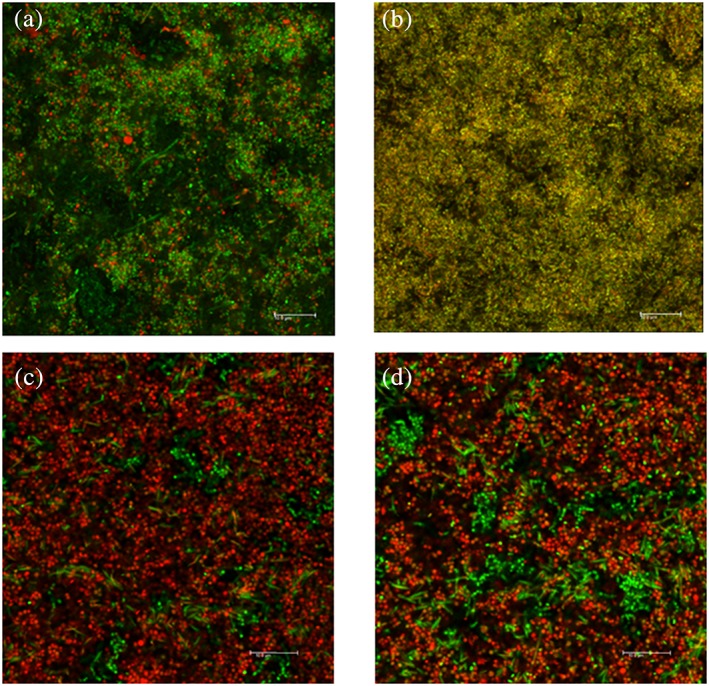
CLSM images of 3‐day old biofilm after antibiotic exposure. Overlapping of images collected from green and red channel. (a) 3‐day‐old biofilm (negative control); (b) 3‐day old biofilm CHX‐treated for 2 hr; (c) 3‐day old biofilm treated with AMX + MET in high concentration for 2 hr; (d) 3‐day old biofilm treated with PV + MET in high concentration for 2 hr. Z‐stacks were taken in xyz projection with 63× objective, oil immersion, at 10 μm from the biofilm bottom. Scale bar: 10 μm

The antibacterial effect of AMX + MET and PV + MET combinations on biofilms was obvious by CLSM imaging, as an increased proportion of red bacterial‐like structures were observed throughout the biofilms' three‐dimensional structure, in contrast to the few remaining green structures, located mainly at the bottom of the biofilms (Figure 4c,d).

## 4 DISCUSSION

A biofilm consisting of three bacterial strains, one known as intermediate colonizer and two as late colonizers in subgingival plaque, was developed in this study. The two late colonizers are periodontal pathogens commonly targeted by systemic antibiotic treatment adjunct to mechanical therapy in periodontal disease (Slots, Research, & Therapy, 2004). In our study, the combination AMX + MET was highly effective against *F*. *nucleatum* and *P*. *gingivalis*, as previously reported in other in vivo and in vitro studies (Astasov‐Frauenhoffer et al., 2014; Sbordone, Barone, Ramaglia, Ciaglia, & Iacono, 1995; Soares et al., 2015). These two anaerobic bacteria may be killed by MET treatment only, in contrast to *A*. *actinomycetemcomitans* that cannot be completely eliminated by MET alone. The exposure of our 3 days biofilm to MET alone resulted in a similar reduction of *A*. *actinomycetemcomitans* but incomplete elimination of the other two species, as live CFU of *F*. *nucleatum* and *P*. *gingivalis* could be retrieved from biofilms treated with MET alone in low concentration (results not shown).

AMX + MET treatment administrated systemically, in individuals who continue to experience loss of periodontal attachment following mechanical debridement, is effective particularly in cases where *A*. *actinomycetemcomitans* is present. The antibiotic combination eliminates or markedly suppresses the periodontal pathogens that remain after subgingival periodontal instrumentation to a level manageable by the host organism, supporting the host defense in overcoming periodontal infections and reducing the risk for recurrent disease progression (Slots et al., 2004).

Infections caused by biofilms are difficult to treat as bacteria in biofilms are 100 to 1,000 times more resistant to antimicrobials compared to the same type of bacteria in a planktonic state (Ceri et al., 1999; Hoiby et al., 2011; Olson, Ceri, Morck, Buret, & Read, 2002). The biofilms thickness increases with the incubation time, containing a higher number of bacteria when reaching the mature state, at 72 hr in our work, as demonstrated in other in vitro studies (Ali Mohammed et al., 2013; Sanchez et al., 2011). The antibiotics might not penetrate the deeper layers of the biofilm and thus may not reach bacteria protected in this environment. The antibiotic concentrations used in this study were very high compared to antibiotic concentration that may be detected in gingival crevicular fluid (Giedrys‐Leeper, Selipsky, & Williams, 1985; Shaddox & Walker, 2009; Tenenbaum, Jehl, Gallion, & Dahan, 1997). However, use of the high concentration antibiotics is counterbalanced by the length of the exposure time, which was much shorter than in other in vitro studies (Belibasakis & Thurnheer, 2014; Soares et al., 2014).

In our study, *A*. *actinomycetemcomitans* was drastically reduced following AMX + MET treatment, both in high and low antibiotic concentrations, but not completely eliminated as in case of *F*. *nucleatum* and *P*. *gingivalis*, despite the high dose antibiotics used. This might be explained by the short exposure time to antibiotics that did not fully affect *A*. *actinomycetemcomitans*, which is more slowly growing. It is also possible that in our model, the nutrients are quickly consumed by the two other species, more rapidly growing, resulting in stationary phase‐like dormant *A*. *actinomycetemcomitans* cells. It is known that antibiotics affect growing cells, thus they are less efficient on bacteria found in a low metabolic activity located in nutrient‐deficient areas in biofilm (Olsen, 2015). Another in vitro study showed that the susceptibility of *A*. *actinomycetemcomitans* in biofilm to six different antibiotics decreases as the biofilm matures (Takahashi, Ishihara, Kato, & Okuda, 2007).

Subgingival strains of *A*. *actinomycetemcomitans* resistant to AMX and MET collected from periodontal patients have previously been reported (Eick, Pfister, & Straube, 1999; Rams, Degener, & van Winkelhoff, 2014b). Because bacterial pathogens resistant both to AMX and MET were found in relatively low frequency (Al‐Haroni, Skaug, & Al‐Hebshi, 2006; Rams et al., 2014b; Rams, Degener, & van Winkelhoff, 2014a), the combination of MET with AMX was recommended rather than using either one alone in order to slow the incidence of developing resistance to MET (Rams et al., 2014a, 2014b).

A multitude of studies have demonstrated clinical effect of administration of the AMX + MET in combination (Sgolastra, Gatto, et al., 2012; Sgolastra, Petrucci, et al., 2012; Zandbergen, Slot, Cobb, & Van der Weijden, 2013). Furthermore, synergistic interactions of AMX and MET on an in vitro multispecies biofilm (Soares et al., 2015) as well as for both combinations, AMX or PV and MET by Etest (Baumgartner & Xia, 2003), have been reported. Pavicic, van Winkelhoff, and de Graaff, 1991 found PG + MET to act synergistically against *A*. *actinomycetemcomitans* in vitro using an agar dilution method and checkerboard titrations. Others found synergy between AMX and MET only on a few of the tested *A*. *actinomycetemcomitans* and *F*. *nucleatum* strains (Kulik Kunz, Lenkeit, Waltimo, Weiger, & Walter, 2014). In our study, PV + MET had similar effect as AMX + MET, by eliminating completely two of the bacterial species from the biofilm and strongly reducing the viable counts of *A*. *actinomycetemcomitans*. The combination PV + MET seemed to be dose‐dependent, as exposure of the biofilms to a three times lower antibiotic concentration resulted in one log higher amount of live CFU retrieved. However, these results should be interpreted with caution, because in our study, the antibiotic concentrations were high, combined with a short exposure time.

It is timely to recall that although even the commensal bacteria in dental biofilm may cause oral pathology, the aim for periodontal treatment is not a complete elimination of resident periodontal species, but to obtain a microbial profile compatible with health by reducing the proportion of pathogenic species (Marsh, Head, & Devine, 2015). A controlled homeostatic immune‐inflammatory state is normal in healthy gingiva, the problems arise when dysbiosis develops with a disturbance in the balance between the host and the microbiota in the biofilm (Lamont & Hajishengallis, 2015).

In this study, CHX 0.2% was used as a positive control. The plaque inhibiting effect and antibacterial properties of chlorhexidine are well documented in vivo and in vitro (Addy, 1986; Schiott et al., 1970). CHX has the ability to penetrate biofilms and having bactericidal effect, acting inside the biofilm (Shapiro, Giertsen, & Guggenheim, 2002). We have used this concentration because with regard to effects on plaque, several reports have demonstrated a significantly better plaque inhibiting effect of 0.2% CHX than 0.12% CHX (Berchier, Slot, & Van der Weijden, 2010; Haydari et al., 2017). However, Soares et al., 2014 found that CHX 0.12% decreased the metabolic activity of the biofilms by approximately 95% and suggested it to be appropriate as a positive control. Regarding clinical effects, most studies do not find statistical significant differences in effect on gingivitis between the concentrations 0.2% and 0.12% (Haydari et al., 2017). To our knowledge, no studies have compared the two CHX concentrations and evaluated the probing pocket depth and/or the periodontal attachment level.

Concluding, in this in vitro biofilm model, the replacement of AMX with PV at the same concentration, in combination with MET, resulted in similar effect on bacterial viability, by eliminating *F*. *nucleatum* and *P*. *gingivalis* and strongly reducing *A*. *actinomycetemcomitans*. The option of using PV + MET instead of AMX + MET deserves further investigation, as this may contribute to reduce the risk of antibiotic resistance development.

## CONFLICT OF INTEREST

The authors have no conflict of interest.
